# Depression and academic achievement in nursing students

**DOI:** 10.1192/j.eurpsy.2026.11691

**Published:** 2026-07-31

**Authors:** M. Efstathiou, A. Grammeniati, Z. Konstanti, M. Kourakos, S. Mantzoukas, M. Gouva, E. Dragioti

**Affiliations:** Department of Nursing, University of Ioannina, Ioannina, Greece

## Abstract

**Introduction:**

Mental health challenges, particularly depression, are highly prevalent among nursing students and have been linked to impaired academic achievement.

**Objectives:**

To examine the relationship between depression and academic achievement among nursing students.

**Methods:**

In this single-centre, observational, cross-sectional study, data were collected from 90 Greek nursing students. The majority were female (82%) with a mean age of 19 years. Depression severity was assessed using the Depressive subscale of the Depression, Anxiety and Stress Scale-21 (DASS-21), while academic achievement was measured both subjectively with the Subjective Academic Achievement Scale (SAAS) and objectively with grade point average (GPA). Descriptive statistics and Pearson’s correlations were calculated.

**Results:**

Analysis of depression severity showed that 28.9% of students reported moderate levels, 11.1% severe, and 7.8% extremely severe. Overall, 47.8% of the sample experienced moderate-to-extremely severe levels of depression. A negative correlation was observed between depression and subjective academic achievement (SAAS) (r=-0.20), whereas the correlation with grade point average (GPA) was weak (r=0.10). Students with severe or extremely severe depression reported the lowest SAAS scores compared to those with normal or mild levels.

**Conclusions:**

Higher levels of depression were linked with lower subjective academic achievement, while no clear association was found with GPA. These findings suggest that depression mainly affects how students perceive their academic performance, which may influence their motivation and engagement.

**Disclosure of Interest:**

None Declared

